# Phytochemical Analysis, GC-MS Chemical Profiling, and In Vitro Antidiabetic Evaluation of South African *Momordica balsamina* Linn Leaf Extracts and Its Effects on Oxidative Stress Modulation

**DOI:** 10.3390/cimb48050503

**Published:** 2026-05-13

**Authors:** Buang Matseke, Daniel Tswaledi, Kokoette Bassey

**Affiliations:** 1 Department of Pharmaceutical Sciences, School of Pharmacy, Sefako Makgatho Health Sciences University, Ga-Rankuwa, Pretoria 0204, South Africa; 2 Department of Biochemistry and Biotechnology, School of Science and Technology, Sefako Makgatho Health Sciences University, Ga-Rankuwa, Pretoria 0204, South Africa; lefatswaledi@gmail.com

**Keywords:** diabetes mellitus, *Momordica balsamina*, oxidative stress, enzyme inhibition, bioactive compounds, cytotoxicity

## Abstract

Background: *Momordica balsamina* L. is widely used in traditional medicine for the management of diabetes in South Africa and globally. This study evaluated the in vitro antidiabetic and cytotoxic effects of *M. balsamina* leaf extracts and identified bioactive compounds potentially responsible for its activity. Methods: Leaves were sequentially extracted using solvents of increasing polarity. Phytochemical composition was determined using standard colorimetric assays, while gas chromatography–mass spectrometry (GC–MS) was employed for compound identification. Antioxidant activity was evaluated using dot blot, DPPH radical scavenging, hydrogen peroxide scavenging, and ferric reducing power assays. Antidiabetic potential was assessed using α-amylase, α-glucosidase, and β-glucosidase inhibitory assays, with acarbose as the reference drug. Cytotoxicity was determined by using the MTT assay on Vero and HEK-293 cell lines. Results: Phytochemical screening revealed alkaloids, flavonoids, terpenoids, saponins, glycosides, and steroids. GC–MS analysis identified compounds associated with antidiabetic activity, including vanillin, 2,4-di-tert-butylphenol, oleic acid, phytol, and hexadecenoic acid. All extracts exhibited antioxidant activity, with the ethyl acetate extract showing the strongest effect. Enzyme inhibition was concentration dependent. The dichloromethane and ethyl acetate extracts showed stronger α-amylase inhibition (IC_50_ = 0.149 and 0.146 mg/mL) than acarbose (0.209 mg/mL). For α-glucosidase, acarbose showed the highest activity, while extracts displayed moderate inhibition. In β-glucosidase assays, both extracts were more active than acarbose. Both extracts were non-cytotoxic up to 500 µg/mL. Conclusions: These findings support the traditional use of *M. balsamina* and highlight its potential as a safe source of antidiabetic agents, warranting further investigation.

## 1. Introduction

Diabetes mellitus is a prevalent metabolic disorder characterized by chronic hyperglycemia resulting from defects in insulin secretion, insulin action, or both [1,2,3]. Persistent hyperglycemia is associated with the development of both microvascular and macrovascular complications, significantly increasing the risk of morbidity and mortality [4,5,6]. The available antidiabetic drugs do not cure diabetes, and they are associated with adverse effects, high costs, and limited availability, particularly in low-resource settings [7,8].

The growing incidence and associated complications of diabetes highlight the urgent need for effective prevention, management, and treatment strategies. As a result, there is growing interest in exploring alternative antidiabetic therapies derived from natural sources, particularly medicinal plants, which offer potential advantages in terms of affordability, accessibility, and safety [9,10]. To date, approximately 1200 plant species have been documented for their traditional use in diabetes management, yet only 30% have been subjected to rigorous biological evaluation [11]. Many of these plants exhibit promising antidiabetic properties, with mechanisms of action that include the inhibition of key carbohydrate-hydrolyzing enzymes such as α-amylase, α-glucosidase, and β-glucosidase [12]. While α-glucosidase catalyzes the final hydrolysis of disaccharides into monosaccharides, primarily glucose, β-glucosidase hydrolyzes cellulose into glucose to facilitate its absorption in the small intestine. α-amylase catalyzes the early breakdown of polysaccharides into disaccharides. The activity of these enzymes significantly contributes to postprandial hyperglycemia, a significant factor in the progression of diabetes and its complications [13,14]. Given their crucial role in carbohydrate metabolism, these enzymes have emerged as key targets for therapeutic intervention; therefore, inhibiting them can effectively slow carbohydrate digestion and thus reduce postprandial glucose spikes and improve glycemic control in diabetic individuals [15]. Consequently, the development of innovative plant-based antidiabetic treatments with improved safety and effectiveness profiles may be facilitated by medicinal plants that can block these enzymes.

Oxidative stress, on the other hand, has been implicated in the development and progression of type 2 diabetes mellitus, as well as its associated complications [16]. Hyperglycemia increases oxidative stress, which in turn contribute to the impairment of pancreatic β-cell function resulting in reduced insulin secretion and action [17]. Antioxidants has been reported to protect the cellular components from oxidative damage, such as protection of β-cell integrity, and thus enhance insulin sensitivity and alleviate diabetes-associated complications [18]. The evaluation of antioxidant activity is therefore highly relevant in antidiabetic studies, particularly when assessing medicinal plants traditionally used for diabetes management.

*Momordica balsamina* Linn (balsam apple), a medicinal plant belonging to the family *Cucurbitaceae*, is widely utilized in the traditional medicine for the treatment and management of various ailments, including diabetes [19,20]. Despite its widespread ethnomedical use, there are a few studies that report antidiabetic properties of the South African species. Furthermore, to ensure the safety of these in-extract bioactive components for therapeutic use, their potential cytotoxicity should be assessed. Most studies have reported the liquid chromatography-mass spectrometry (LC-MS) of *M. balsamina* leaf extracts, therefor, to add to this existing data, gas chromatography–mass spectrometry (GC-MS) analysis has been employed to identify other phytochemical constituents of *M. balsamina*, revealing a diverse array of bioactive compounds that may contribute to its antidiabetic activity. The purpose of this study is to evaluate the in vitro antioxidant and antidiabetic potential of *M. balsamina* extracts using enzyme inhibition assays, assess their cytotoxicity using the (3-(4,5-dimethylthiazol-2-yl)-2,5-diphenyltetrazolium bromide) tetrazolium reduction (MTT) assay, and correlate these biological activities with the bioactive compounds identified via GC-MS analysis. The findings of this study will add to the expanding corpus of research on plant-derived antidiabetic drugs and provide important new insights into the possible pharmacological uses of *M. balsamina*.

## 2. Materials and Methods

### 2.1. Materials and Reagents

All the chemicals and reagents used in this study were of analytical grade. Alpha-glucosidase, alpha-amylase, beta-glucosidase, 2-napthyl-beta-D-glucopyranoside, acarbose, glucose, Vero cell lines, Dulbecco’s Modified Eagle Medium (DMEM), and fast blue salts were obtained from Sigma-Aldrich (Darmstadt, Germany).

### 2.2. Plant Material

Fresh leaves of *M. balsamina* were collected from Phake ya Ratlhagane village (25°08′51.1″ S, 28°30′28.2″ E) in Mpumalanga Province, South Africa, on November 2022 with the assistance of an indigenous health practitioner. Plant specimens were taken to the South African National Biodiversity Institute (SANBI) for botanical identification. A voucher specimen MB001 was then deposited in the Department of the Pharmaceutical Sciences, School of Pharmacy, Sefako Makgatho Health Sciences University. The plant materials were air-dried in a well-ventilated area away from direct sunlight to prevent degradation of bioactive compounds. During and after the drying process, seeds were carefully removed from the fruit pulp. The dried samples were then finely ground using a Polymix Laboratory Dry Mill Drive Unit (Polymix™ PX-MFC 90 D, Kinematica AG, Luzern, Switzerland) and stored in airtight containers at room temperature in a laboratory cupboard until further analysis.

### 2.3. Preparation of the Extracts

The dried leaves were ground into a fine powder (0.91560 kg) and sequentially extracted using solvents of increasing polarity, namely hexane, dichloromethane, ethyl acetate, and methanol, starting from the least polar (hexane) to the most polar (methanol). The extraction was performed in 24 h cycles, with two repetitions on an orbital platform shaker. The resulting extracts were filtered and concentrated by evaporating the solvents using a Stuart rotary evaporator (RE400, Cole-Parmer Ltd., Stone, ST15 OSA, UK) at 37 °C and a speed of 120 rpm. The concentrated extracts were further dried under a stream of air at room temperature. After determining the weight of the dried extracts, they were subsequently stored at room temperature until further use.

### 2.4. Phytochemical Screening

#### 2.4.1. Qualitative Phytochemical Screening

*M. balsamina* leaf extracts were subjected to preliminary phytochemical tests to determine either the presence or absence of alkaloids, flavonoids, saponins, anthraquinones, terpenoids, glycosides, steroids, and tannins. The phytochemical tests reported by [21,22] were followed with minor modifications.

#### 2.4.2. Quantitative Phytochemical (TPC and TFC) Screening of *Momordica balsamina* Linn Leaf Extracts

##### Determination of Total Phenolic Content (TPC)

The total phenolic content was determined using the Folin–Ciocalteu reagent method as described by [23] with modifications. A calibration curve was constructed using gallic acid standard solutions at concentrations of 20, 40, 60, 80, and 100 µg/mL. For each standard, 1 mL of gallic acid solution was transferred into separate 25 mL volumetric flasks containing 9 mL of distilled water. Subsequently, 1 mL of Folin–Ciocalteu reagent was added to each flask. The mixtures were shaken thoroughly and allowed to stand at room temperature for 5 min. Thereafter, 10 mL of 7% sodium carbonate solution was added, and the volume was adjusted to 25 mL with distilled water. The reaction mixtures were incubated for 90 min at room temperature, after which the absorbance was measured at 550 nm using a Nanocolor UV/VIS 2 spectrophotometer (Macherey-Nagel GmbH & Co. KG, Wertheim, Germany). Crude extract samples were prepared following the same procedure as the standards, each in triplicate, and their absorbance values were recorded at the same wavelength. The total phenolic content was expressed as micrograms of gallic acid equivalent per grams of extract (µg GAE/g extract).

##### Determination of Total Flavonoid Content (TFC)

The total flavonoid content of the plant was determined using the aluminum chloride colorimetric method, as described by [23] with slight modifications. A calibration curve was constructed using quercetin standard solutions at concentrations of 20, 40, 60, 80, and 100 µg/mL. For each standard, 1 mL of quercetin solution was added to separate test tubes, followed by 3 mL of methanol. Thereafter, 0.2 mL of 10% aluminum chloride and 0.2 mL of 1 M potassium acetate were added to the mixture, followed by 5.6 mL of distilled water. The reaction mixtures were incubated at room temperature for 30 min, after which the absorbance was measured at 420 nm using a Macherey-Nagel Nanocolor UV/VIS 2 spectrophotometer (Wertheim, Germany). The total flavonoid content was calculated from the calibration curve and expressed as micrograms of quercetin equivalent per grams of extract (µg QE/g extract).

### 2.5. Gas Chromatography–Mass Spectrometry (GC-MS) Analysis

The phytochemical constituents of *M. balsamina* extracts were analyzed using GC-MS. The analysis was conducted using a Shimadzu QP2010 SE (Shimadzu Corporation, Kyoto, Japan) gas chromatograph–mass spectrometer, equipped with an inert cap 5MS/SIL silica capillary column (30 m × 0.25 mm ID × 0.25 µm df), and comprising 100% dimethylpolysiloxane. Helium (99.99%) was used as the carrier gas at a constant flow rate of 1 mL/min. A 2 µL sample was injected with a split ratio of 1:10. The injector temperature was set to 290 °C, and the ion source temperature was maintained at 230 °C. The oven temperature program was as follows: isothermal at 50 °C for 1 min, increased to 180 °C at 20 °C/min, held at 180 °C for 5 min, then increased to 240 °C at 5 °C/min, and finally increased to 280 °C at 20 °C/min. The mass spectra were recorded at 70 eV with a scan interval of 0.3 s over a mass range of 50–700 *m*/*z*. Data acquisition and analysis were performed using GC-MS Solutions software, version 2.6. Compound identification was achieved by comparing the obtained mass spectra with those in the National Institute of Standards and Technology (NIST) library and by analyzing retention indices. The relative percentage of each compound was determined by calculating the peak area normalization.

### 2.6. Antioxidant Activity

#### 2.6.1. Dot Plot Method

The TLC dot plot method described by [24] was applied with minor modifications. The dry leaf extracts were redissolved in the solvents that were used to extract them. The TLC plate, with pre-coated silica gel 60 and fluorescent indicator UV_254_, was prepared in such a way that it accommodated the plotting of all the eight extracts. Each extract was spotted in a dot form on the TLC plate, and the plate was then left to dry under a stream of air in the laboratory for five minutes. The TLC plate was then sprayed with the prepared 0.2 mM of 2,2-diphenyl-1-picrylhydrazyl (DPPH) solution. The white to pale yellow discoloration on the purple background of the TLC plate was taken as a positive indication of antioxidant activity of the plant extracts.

#### 2.6.2. DPPH Antioxidant Activity Assay

The DPPH radical scavenging activity of the plant extracts was determined following the methods described by [22,25] with a few modifications. Standard solutions of various concentrations (0.2, 0.4, 0.6, 0.8, and 1.0 mg/mL of the extracts), as well as 0.8 mM of DPPH in methanol, were prepared. For the test, 1.0 mL of each prepared concentration were transferred into a test tube and 1.0 mL DPPH solution was then added to it. The mixture was vortexed and then incubated for 30 min at room temperature. Thereafter, 200 µL of each of the mixtures were transferred into separate wells of a 96-well plate and their absorbances were measured at 517 nm against the blank. BHT and gallic acid solutions were prepared using methanol in a ratio of 1 mg/mL, and different concentrations were prepared as the samples and used as positive control standards. The ability of the plant extracts to scavenge free radicals was calculated using the equation below.

$$\%\;DPPH\;radical\;scavenging\;activity=\frac{A_{control}-A_{sample}}{A_{control}}\times 100$$

where A_sample_ = absorbance of the sample, and A_control_ = absorbance of the negative control.

#### 2.6.3. Hydrogen Peroxide Activity Assay

The ability of *M. balsamina* leaf extract to scavenge hydrogen peroxide (H_2_O_2_) was performed using the method described by Olivier and colleagues [22] with minor modifications. Different concentrations (0.2, 0.4, 0.6, 0.8, and 1.0 mg/mL) of the extracts were prepared, and 1.0 mL of each prepared concentration was transferred into a test tube. To each of the extract solutions in the test tubes, a 2 mL solution of 40 mM phosphate buffer and distilled water (pH 7.4) was added. The reaction mixture was vortexed at 3000 revolutions per minute (rpm) and then incubated for 10 min at room temperature. In total, 200 µL of each mixture was transferred into a well on a 96-well plate (Eppendorf® Microplate 96/U-PP, 96 well, Sigma-Aldrich) and their absorbance were measured at 560 nm against a blank solution, in this case methanol. Gallic acid and BHT solutions were used as positive control standards. The ability of the extracts to scavenge H_2_O_2_ was calculated using the equation below.

$$\%\;H_{2}O_{2}\;radical\;scavenging\;activity=\frac{A_{control}-A_{sample}}{A_{control}}\times 100$$

where A_control_ = absorbance of the negative control, and A_sample_ = absorbance of the sample.

#### 2.6.4. Ferric Reducing Antioxidant Power (FRAP) Assay

To determine the reducing power ability of the plant extracts, a method described by Olivier and colleagues [22] was followed with minor modifications. *M. balsamina* hexane, DCM, EA, and MeOH extracts were redissolved in the solvents used to extract them at a concentration of 1.0 mg/mL. Different concentrations (0.2, 0.4, 0.6, 0.8, and 1.0 mg/mL) of the extracts were prepared. In total, 1.0 mL of each concentration was transferred into test tubes. To each test tube, 2.5 mL of 1.0% potassium ferricyanide (K_3_Fe(CN)_6_) and 2.5 mL of 0.2 M sodium phosphate buffer (pH 6.6) were added. The reaction mixture was vortexed at 3000 rpm and then incubated at 50 °C for 30 min. Following that, 2.5 mL of 10% trichloroacetic acid (TCA) was added and centrifuged at 3000 rpm for 10 min. In total, 2.5 mL of the supernatant was mixed with 2.5 mL of deionized water and 0.5 mL of 0.1% ferric chloride. The absorbance of each mixture was measured at 700 nm against blank methanol. BHT and gallic acid were used as positive standard controls, and they were prepared in the same manner as the extracts. The reducing power ability of the samples was determined using the equation below.

$$\%\;Reducing\;power\;activity=\frac{A_{control}-A_{sample}}{A_{control}}\times 100$$

where A_sample_ = absorbance of the sample, and A_control_ = absorbance of the negative control.

### 2.7. Antidiabetic Activity

#### 2.7.1. Inhibition Assay for α-Amylase Activity

A modified version of the Poovitha and Parani [26] method was used to assess the α-glucosidase inhibitory activity of *M. balsamina* extracts and compounds. The re-constituted *M. balsamina* extracts were serially diluted throughout a 96-well microplate after 50 μL of potassium phosphate buffer (pH 7.0) was added to each well. Each well was then filled with 100 μL of a commercial glucose test reagent and 50 μL of a sucrose solution. An aliquot of 30 μL of intestinal acetone rat powder was added to each well to start the reaction, and the plate was then incubated for 30 min at 37 °C. The same process was used to make acarbose, which was utilized as a positive control. A Molecular Devices^®^ microplate reader was used to measure the absorbance at 505 nm. The percentage inhibition of α-glucosidase activity was calculated using the equation below.

$$\%\;\alpha -amylase\;inhibition=\frac{A_{0}-A_{S}}{A_{0}}\times 100$$

where A_s_ is the absorbance in the presence of the test substance, and A_0_ is the absorbance of the control.

#### 2.7.2. Inhibition Assay for α-Glucosidase Activity

A modified α-glucosidase assay described by Bhatia and colleagues [27] was used to assess the α-glucosidase inhibitory activity of *M. balsamina* extracts. The produced *M. balsamina* extracts were serially diluted throughout a 96-well microplate after 50 μL of potassium phosphate buffer (pH 7.0) was added to each well. Each well was then filled with 100 μL of a commercial glucose test reagent and 50 μL of a sucrose solution. An aliquot of 30 μL of intestinal acetone rat powder was added to each well to start the reaction, and the plate was then incubated for 30 min at 37 °C. The same process was used to make acarbose, which was utilized as a positive control. A spectrophotometer was used to detect absorbance at 505 nm. The percentage inhibition of α-glucosidase activity was calculated using the below equation, and the IC_50_ extracts were also determined using linear regression equations.

$$\%\;\alpha -glucosidase\;inhibition=\frac{A_{0}-A_{S}}{A_{0}}\times 100$$

where A_s_ is the absorbance in the presence of the test substance, and A_0_ is the absorbance of the control.

#### 2.7.3. Inhibition Assay for β-Glucosidase Activity

The β-glucosidase inhibitory activity of *M. balsamina* extracts was assessed at varying concentrations (0.2, 0.4, 0.6, 0.8, and 1.0 mg/mL). In total, 120 µL of each concentration was added to a 96-well microplate, followed by the addition of β-glucosidase enzyme. The plate was incubated at 37 °C for 15 min. Subsequently, 20 µL of 2-Naphthyl-β-D-glucopyranoside was added to each well, and the plate was incubated for an additional 15 min at 37 °C. The reaction was terminated by adding 80 µL of sodium carbonate solution to each well. Absorbance was measured at 405 nm using a spectrophotometer. Acarbose, prepared under similar conditions, served as the positive control. The percentage inhibition of β-glucosidase activity was calculated using the equation below. The IC_50_ of the extracts were also determined using linear regression equations [28].

$$\%\;\beta -glucosidase\;inhibition=\frac{A_{0}-A_{S}}{A_{0}}\times 100$$

where A_s_ is the absorbance in the presence of the test substance, and A_0_ is the absorbance of the control.

### 2.8. Cytotoxicity

#### 2.8.1. Preparation of the Cell Lines

The Vero and HEK-293 cell lines (American Type Culture Collection [ATCC], Manassas, VA, USA) were maintained, cultured in Dulbecco’s Modified Eagle Medium (DMEM) (Sigma-Aldrich, Johannesburg, South Africa) and supplemented with 10% fetal bovine serum (FBS) and 1% penicillin–streptomycin (PSN). The cells were incubated at 37 °C in a 95% humidified environment containing 5% CO_2_. The culture medium was replaced with fresh medium every 2–3 days until the cells reached 70–80% confluency. Using a light microscope (Nikon TS100, Schönwalde-Glien, Germany), cell morphology was regularly observed to evaluate cell viability, attachment, mycoplasma contamination, and any morphological alterations. Cell counting was performed using Cell Countess (Thermofisher, Waltham, MA, USA), at a 1:1 cell to trypan blue dye ratio.

#### 2.8.2. 3-(4,5-Dimethylthiazol-2-yl)-2,5-diphenyltetrazolium Bromide (MTT) Cytotoxicity Assay

The MTT test was used to evaluate the cytotoxicity of viable cells after treatment with different concentrations of *M. balsamina* extracts in vitro, as described by [29] with minor modifications. The process was started by seeding the cell suspension (2500 cells/mL) into 96-well microplates for 24 h at 37 °C in a humidified environment with 5% CO_2_. This was performed to allow cell adhesion to take place and achieve cell confluency. After 24 h of incubation, a TC20 cell counter (Bio-Rad, Hercules, CA, USA) was used to calculate the number of cells needed for the experiment. Following attachment, cells were treated with different concentrations of dichloromethane and ethyl acetate extracts (1, 0.5, 0.25, and 0.125 mg/mL) and further incubated for 24 h under the same conditions. After incubation, the cells were treated with 20 µL of MTT reagent (5 mg/mL in PBS) (Sigma-Aldrich, Johannesburg, South Africa) and the plates were incubated for an additional 4 h. The resulting yellow formazan crystals were dissolved in 100 µL of dimethyl sulfoxide (DMSO). The negative control was the untreated cells, whereas the positive control was hydrogen peroxide. The absorbance of the formazan product was measured at 560 nm using a GloMax-Multi microplate reader (Promega, Madison, WI, USA). The percentage of cell viability was calculated using the formula below.

$$Cell\;viability\left(\%\right)=\frac{Average\;OD\;(experimental\;group)}{Average\;OD\;(untreated\;group)}\times 100$$

Data for concentration response curves and statistical analysis (one-way ANOVA) were executed using GraphPad Prism^®^ version 8.4.2, GraphPad Software Inc., San Diego, CA, USA.

## 3. Results and Discussion

### 3.1. Qualitative Phytochemical Screening

The qualitative phytochemical analysis of *M. balsamina* leaf extracts revealed the presence of alkaloids, flavonoids, saponins, terpenoids, glycosides, steroids, and tannins (Table 1) in varying degrees depending on solvent polarity. Anthraquinones were the only compounds that were absent in all the tested extracts, suggesting that they did not contribute to the biological activities of this plant. Most of these phytochemicals have been reported to contribute to the antidiabetic compounds of several medicinal plants. Alkaloids were observed in all the extracts and strongly in the polar ethyl acetate and methanol extracts. Alkaloids have the ability to inhibit carbohydrate digesting enzymes such as α-glucosidase. They do so by binding to the active sites of these enzymes, causing a distraction to the formation of enzyme–substrate complex and thus reducing the enzyme activity [30]. Dietary flavonoids have mostly beneficial antidiabetic properties, and they were consistently present in all the extracts. They have been reported to improve diabetes and its complications through the regulation of glucose metabolism and enzyme activities [31]. The results also revealed the presence of saponins in all the extracts but strongly present in methanol extracts. This class has been associated with lowering of hypoglycemic activity by enhancing insulin release and reducing intestinal glucose absorption [32]. Tannins that were found strongly on the polar ethyl acetate and methanol have also been reported to play a role in the prevention and management of diabetes and its associated complications; however, the mechanism of action is not yet documented [33].

Overall, the presence and distribution of these phytochemicals, particularly in the ethyl acetate and methanol extracts, suggest that the plant is a rich source of bioactive compounds with potential antidiabetic properties. The coexistence of flavonoids, terpenoids, tannins, glycosides, and saponins indicates the possibility of complementary or synergistic mechanisms, such as the modulation of glucose metabolism, antioxidant effects, and enzyme inhibition. 

**Table 1:** Preliminary phytochemical screening results of *Momordica balsamina* leaf extracts.

Phytochemical Test	Observation for Different Solvent Extracts			
	Hexane	Dichloromethane	Ethyl Acetate	Methanol
Alkaloids	+	+	++	++
Flavonoids	+	+	+	+
Saponins	+	+	+	++
Anthraquinones	-	-	-	-
Terpenoids	+	+	++	++
Glycosides	+	++	+	+
Steroids	+	-	+	+
Tannins	+	+	++	++

Key: - absent, + present, ++ strongly present.

### 3.2. Quantitative Phytochemical (TPC and TFC) Screening of Momordica balsamina Leaf Extracts

Quantitative phytochemical analyses of phenolic compounds and flavonoids were performed, supporting the qualitative findings by highlighting the contribution of key antidiabetic compounds across the different extracts. Only the total phenolic and flavonoid contents were quantified in this study because these classes of phytochemicals are widely recognized as the primary contributors to antidiabetic and antioxidant activities in medicinal plants. The total phenolic content (TPC) was highest in the dichloromethane extract (16.553 ± 1.221 µg/mL), suggesting a strong potential for enzyme inhibition and antioxidant activity, both of which are important in managing hyperglycemia and oxidative stress in diabetes mellitus. The total flavonoid content (TFC), on the other hand, was most abundant in the methanol extract (4.889 ± 0.060 µg/mL) Table 2, and was consistent with the qualitative observation of a richer presence of polar bioactive compounds in this extract. Flavonoids are known to enhance insulin secretion, improve glucose uptake, and inhibit carbohydrate-digesting enzymes, indicating that the methanol extract may possess superior antidiabetic potential. Overall, the distribution of phenolics and flavonoids across the extracts reinforce their collective role in contributing to the plant’s antidiabetic activity.

### 3.3. Phytochemical Characterization M. balsamina Leaf Extracts by GC-MS Analysis

**Figure 1:**
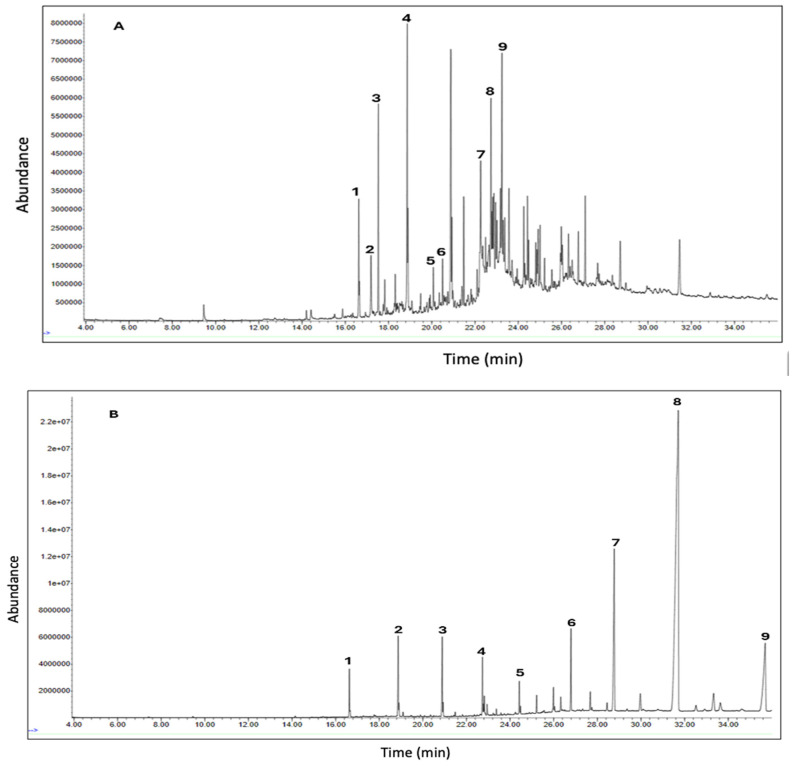
GC-MS chromatograms of DCM (**A**) and hexane (**B**) extracts of *M. balsamina* leaves.

**Figure 2:**
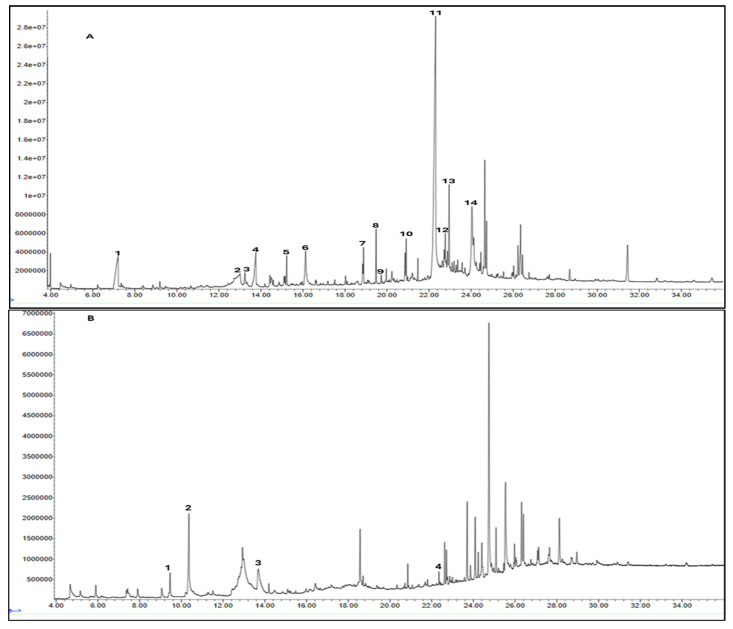
GC-MS chromatograms of EA (**A**) and MeOH (**B**) extracted from the leaves of *M. balsamina*.

GC-MS was used to identify the phytochemicals potentially responsible for the plant extracts’ antidiabetic properties. The results of GC-MS analysis revealed nine distinct compounds in the DCM extract, nine in the hexane extract (Figure 1 and Table 3), fourteen in the EA extract, and four in methanol extract (Figure 2 and Table 4). Notably, several of these compounds have been previously reported to exhibit antidiabetic properties, suggesting their potential contributions to the observed antioxidant and antidiabetic potentials of *M. balsamina*. However, several other peaks in all the extracts could not be identified due to zero matches in the GC-MS library.

#### 3.3.1. GC-MS Analysis of DCM and Hexane Leaf Extracts

Amongst the compounds in the DCM extracts were hexadecane, vanillin, 2,4-di-tert-butylphenol, octadecane, eicosane, hexadecenoic acid ethyl ester, hexadecenoic acid, hexadecenoic acid butyl ester, and decanol-2-hexyl (Figure 1 and Table 3). As for the hexane extract, all the identified compounds were saturated with hydrocarbons hexadecane, octadecane, eicosane, docosane, tetracosane, heptacosane, octacosane, tetrtricotane, and heptacosane. Table 3 reports the proposed antidiabetic compounds from the dichloromethane and hexane extracts. A study by [34] demonstrated that vanillin inhibited α-glucosidase enzyme activity, with the inhibitory effect positively correlated with vanillin concentration. Vanillin was successfully bound to α-glucosidase with a binding free energy of −8.42 Kcal/mol, indicating good binding (−7 to −9 Kcal/mol), and this suggested good potential for managing postprandial hyperglycemia, a key factor in controlling type 2 diabetes. These results provide evidence for vanillin’s role in inhibiting carbohydrate digestion and absorption, thereby lowering postprandial blood glucose levels.

The compound 2,4-di-tert-butylphenol has been reported to inhibit α-amylase activity, suggesting it may contribute to the antidiabetic properties of the DCM extract. This compound has been shown to have a high binding affinity for human pancreatic α-amylase, suggesting its role in inhibiting starch breakdown and subsequent glucose absorption. The compound further displayed improved metabolism, with a skin permeability of −3.87 cm/s, gastrointestinal absorption of 95.48%, and a total clearance of 0.984 log mL min^−1^ kg^−1^, indicating a promising pharmacokinetic profile and bioavailability [35].

In the present study, vanillin (peak 2, Figure 1A) helps reduce postprandial glucose levels by inhibiting α-glucosidase, whereas 2,4-di-tert-butylphenol (peak 3, Figure 1A) modifies starch digestion by decreasing α-amylase activity. These findings therefore suggest a synergistic interaction of the two compounds identified in the DCM extract. The dual action of the two compounds supports the promising potential of the DCM extract as a successful therapeutic strategy for the management of diabetes and hyperglycemia. In addition, vanillin’s antioxidant qualities and 2,4-di-tert-butylphenol’s anti-inflammatory effects may enhance their antidiabetic action by reducing inflammation and oxidative stress, which can worsen insulin resistance in people with diabetes.

GC-MS analysis of the hexane leaf extract revealed the presence of various long-chain alkanes. Of all the alkanes identified, only one compound has been reported to influence the antidiabetic properties of a plant extract directly. A study by Fayez and colleagues (2022) [36] demonstrated that the compound t tetratriacontane (peak 8, Figure 1B) can inhibit the activity of α-amylase and α-glucosidase, supported by molecular docking with the binding energy of −7 kcal/mol for α-amylase and −12.1 kcal/mol for α-glucosidase, indicating moderate to good binding affinity. The two enzymes play separate roles in lowering the postprandial hyperglycemia, a therapeutic target in the management of diabetes [37]. The absence of antidiabetic activity in the other identified compounds might suggest that they have not yet been reported or they do not contribute to the extract’s antidiabetic activity.

**Table 3:** GC-MS phytochemical profiling of DCM and hexane extracts of *M. balsamina* leaves. The structures in the table were drawn by the authors using ChemDraw Ultra 8.0 (Chemistry Software Ltd., Gateways, Surrey, UK).

Peak No	RT (min)	Compound Name	Compounds Structure	Composition % Area
**DCM Extract Chemical Characterization**				
1	16.624	Hexadecane	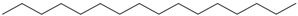	2.551
2	17.191	Vanillin	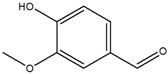	1.873
3	17.528	2,4-di-tert-butylphenol	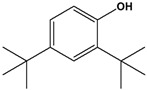	3.859
4	18.865	Octadecane	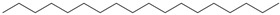	7.831
5	20.878	Eicosane	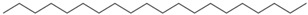	9.559
6	21.479	Hexadecenoic acid, ethyl ester	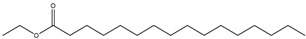	3.461
7	22.260	n-Hexadecenoic acid	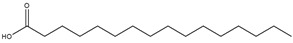	14.304
8	23.245	Hexadecenoic acid, butyl ester	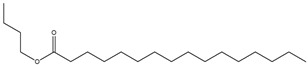	42.822
9	2372	1-Decanol, 2-hexyl	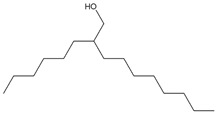	5.430
**Hexane Extract Chemical Characterization**				
1	16.633	Hexadecane	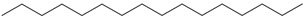	1.673
2	18.865	Octadecane	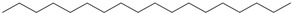	2.893
3	20.883	Eicosane	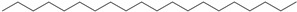	3.246
4	22.732	Docosane	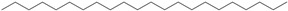	3.093
5	24.422	Tetracosane	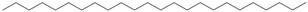	1.700
6	26.790	Heptacosane	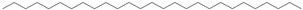	4.176
7	28.764	Octacosane	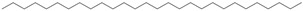	13.018
8	31.666	Tetratriacotane	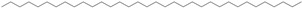	52.129
9	35.669	Heptacosane	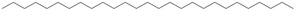	9.201

#### 3.3.2. GC-MS Analysis of EA and MeOH Leaf Extracts

The EA extract consisted of a combination of straight, branched, and ring alcohols as phenols, amines, and certain alkanoic acids. Amongst these compounds were propylene glycol, glycerin, and 2,3-dihydro-3,5-dihydro-6-methyl-4-pyrone. The others were 1,2,3-propanetriol-1-acetate; 2-methoxy-4-vinylphenol; 2-methyl-4-amino-6-dimethylamino-s-triazine; 1-nonadecene; neophytadiene; 3,7,11,15-Tetramethyl-2-hexadecen-1-ol; hexadecenoic acid ethyl ester; n-hexadecenoic acid; oleic acid; phytol; and octadecanoic acid.

Table 4 shows the presence of propylene glycol (peak 1, Figure 2A) in the EA extract, and this compound has been reported to possess a α-glucosidase inhibitory effect. Propylene glycol exhibited concentration-dependent α-glucosidase inhibition activity, with a maximum inhibition of 87.79 ± 1.86% at 800 µg/mL [38]. A study by Mhya and colleagues (2021) [39] demonstrated that the α-α-amylase-vi α-β-glucosidase enolic compound can inhibit both α-amylase and α-glucosidase activities. The study further performed molecular docking against these two enzymes. The compound exhibited binding energies of −4.4 kcal/mol (α-amylase) and −4.2 kcal/mol (α-glucosidase) against the standard drug acarbose with −3.9 (α-amylase) and −4.8 kcal/mol (α-glucosidase), indicating moderate binding affinity [40]. Another compound, phytol (peak 13, Figure 2A), may contribute to the antidiabetic effect of the EA extract, showing a strong binding interaction with the protein PPAR-γ (binding energy of −7.27 kcal/mol), whose upregulation has been reported to alleviate insulin resistance and glucose uptake, thereby reducing blood glucose levels [41].

Octadecanoic acid (peak 14, Figure 2A), commonly known as stearic acid, has been reported to inhibit protein tyrosine phosphatase 1B (PTP1B) activity [35]. As a potential PTP1B inhibitor, stearic acid may enhance insulin receptor signaling, thereby increasing glucose uptake in adipocytes [42]. As another fatty acid, oleic acid has also been reported to improve glucose uptake by enhancing insulin receptor signaling and increasing IRS1 expression in a dose-dependent manner [43]. GC-MS results for the EA leaf extract revealed the presence of bioactive compounds that inhibit key carbohydrate-digesting enzymes, such as α-amylase and α-glucosidase. The inhibition of these enzymes can lead to slow carbohydrate digestion and glucose absorption, thereby regulating postprandial glucose levels. The results also revealed the presence of various fatty acids that modulate proteins involved in the insulin signaling pathway, such as insulin receptor substrate (IRS), phosphatidylinositol 3-kinase (PI3K), and adenosine monophosphate-activated protein kinase (AMPK).

Unexpectedly, the methanol extract demonstrated limited antidiabetic activity across the three assays compared to the other extracts. This suggested that the methanol extract might not contain the phytochemicals that can strongly regulate glucose, as such compounds would have solubilized in the EA prior to the methanol extraction. GC-MS results also identified the fewest compounds in methanol (4) than in hexane (9), DCM (9), and EA (14). Among the four identified compounds, none have been reported to be directly involved in glucose regulation; however, 2-methylhexadecan-1-ol (peak 4, Figure 2B) and cyclohexyl isocyanate (peak 1, Figure 2B) contribute to glucose regulation. Cyclohexyl isocyanate has been used in the synthesis of sulfonylurea derivatives, a class of antidiabetic drugs [44]. The observed results suggest that the methanol extract may contain minor phytochemicals with potential activity, which may be undetectable by GC-MS.

**Table 4:** GC-MS phytochemical profiles of EA and MeOH extracts of *M. balsamina* leaves. The structures in the table were drawn by the authors using ChemDraw Ultra 8.0 (Chemistry Software Ltd. Gateways, Surrey, UK).

Peak No	RT [min]	Compound Name	Compound Structure	Composition % Area
**EA Extract Chemical Characterization**				
1	7.180	Propylene glycol	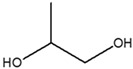	4.679
2	12.991	Glycerin	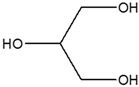	5.022
3	13.240	2,3-Dihydro-3,5-dihydroxy-6-methyl-4-pyrone	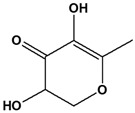	1.368
4	13.741	1,2,3-Propanetriol, 1-acetate	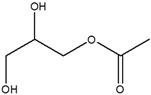	3.097
5	15.227	2-Methoxy-4-vinylphenol	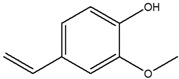	1.627
6	16.123	2-Methyl-4-amino-6-dimethylamino-s-triazine	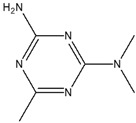	2.906
7	18.877	1-Nonadecene	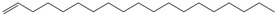	2.33
8	19.480	Neophytadiene	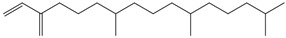	1.53
9	19.962	3,7,11,15-Tetramethyl-2-hexadecen-1-ol	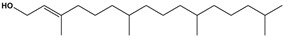	1.057
10	21.469	Hexadecenoic acid ethyl ester	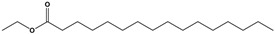	2.211
11	22.293	n-Hexadecanoic acid	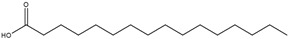	28.554
12	22.764	Oleic acid	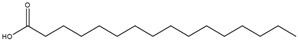	5.567
13	22.953	Phytol	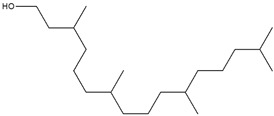	5.741
14	24.043	Octadecanoic acid	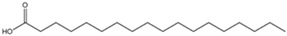	11.823
**Methanol Extract Phytochemical Profile**				
1	9.461	Cyclohexyl isocyanate	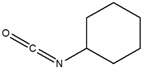	1.625
2	10.365	Neopentyl glycol	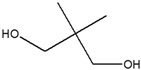	6.648
3	13.696	1,2,3-Propanetriol, 1-acetate	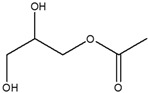	5.813
4	22.622	2-Methylhexadecan-1-ol	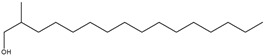	3.16

### 3.4. Antioxidant Activity

#### 3.4.1. Qualitative Antioxidant Analysis

**Figure 3:**
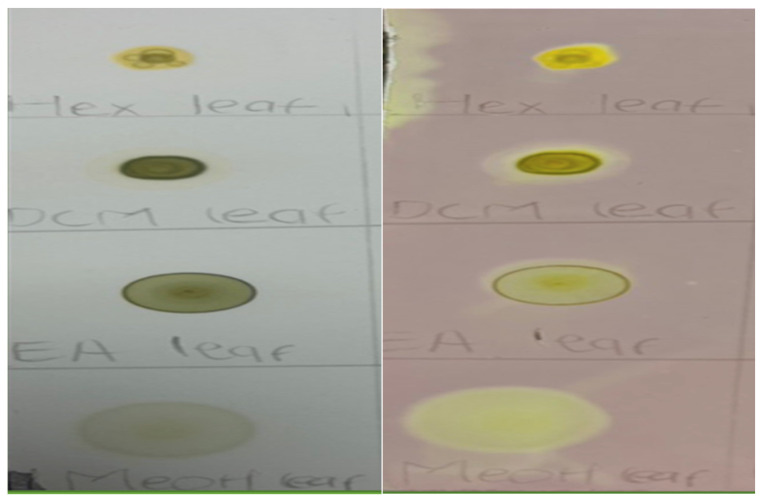
Dot plot of *M. balsamina* leaf extracts before spraying with DPPH (**left**) and after spraying with DPPH (**right**).

To determine the antioxidant activity of *M. balsamina* extracts, each sample was diluted, spotted on the prepared TLC plate, and stained with DPPH solution. The method depended on the ability of the extracts to inhibit the accumulated oxidized products. As depicted in Figure 3, this occurs when the antioxidants, in this case the extracts, donate an electron to the stable free radical (DPPH), which results in the reduced form of DPPH and thus bleaching to yellowish or whitish color of DPPH. All *M. balsamina* leaf extracts exhibited notable antioxidant activity. The phytochemical analysis of *M. balsamina* leaf extracts revealed flavonoid, alkaloid, saponin, steroid, glycoside, and phenolic compounds. These bioactive constituents have been reported to possess antioxidant properties, primarily through their ability to donate hydrogen atoms or electrons to stabilize reactive and unstable free radicals. This free radical scavenging capacity likely accounts for the observed antioxidant profiles of *M. balsamina* leaf extracts.

#### 3.4.2. Quantitative Antioxidant Activity

##### DPPH Radical Scavenging Activity

**Figure 4:**
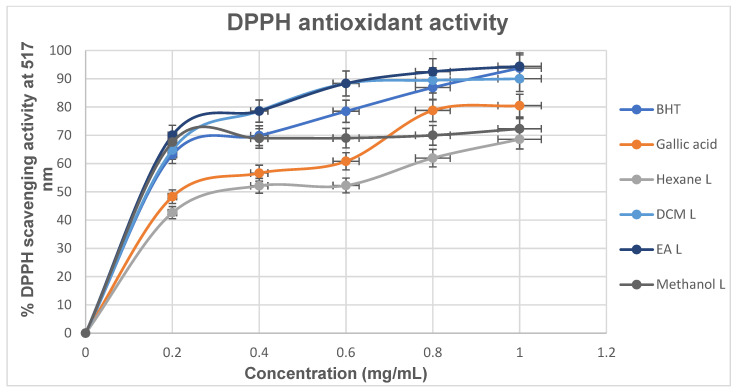
Percentage (%) DPPH radical scavenging activity of *M. balsamina* leaf and fruit extracts of different concentrations.

Figure 4 represents the DPPH radical scavenging activity of *M. balsamina* leaf extracts relative to that of the gallic acid and butylated hydroxytoluene (BHT) standards. All the extracts and standards exhibited a concentration-dependent manner, indicating dose-dependent antioxidant potential. Among the extracts, the ethyl acetate extract showed the highest activity in all the concentrations tested (70.05% to 94.41%), as compared to the standard BHT with a percentage inhibition of 63.20% to 93.22% and gallic acid (48.30% to 80.5%).

##### Hydrogen Peroxide Scavenging Activity

**Figure 5:**
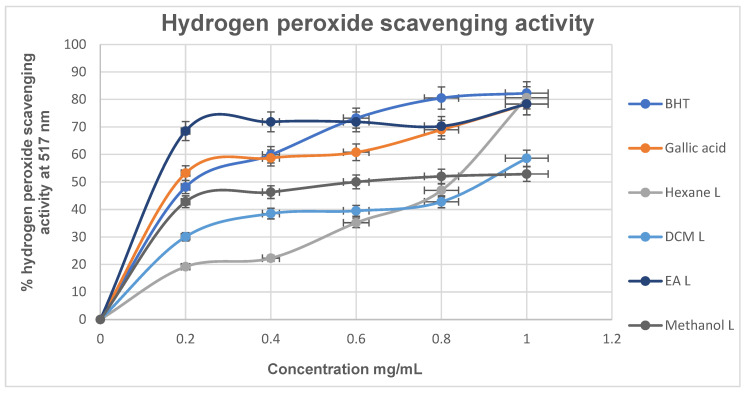
Percentage (%) hydrogen peroxide radical scavenging activity of *M. balsamina* leaf and fruit extracts at different concentrations.

Hydrogen peroxide is a reactive oxygen species (ROS) capable of inactivating various enzymes and is well known for its toxic effects in biological systems [45]. The ability of plant extracts to scavenge H_2_O_2_ can reduce oxidative stress and the associated toxicity, thereby contributing to protection against diseases such as diabetes. As shown in Figure 5, the standard BHT exhibited the highest hydrogen peroxide scavenging activity, with 82.30% inhibition at the highest concentration tested. This was followed by the hexane leaf extract (80.61%). At lower concentrations (0.2–0.6 mg/mL), the moderately polar ethyl acetate (EA) leaf extract demonstrated the highest inhibition (68.51%), exceeding that of BHT (48.16%) and gallic acid (53.22%). This suggests that the EA extract possesses strong hydrogen peroxide scavenging activity, particularly at lower concentrations. Furthermore, the observed effectiveness of polar extracts aligns with traditional medicinal practices, where polar solvents are commonly used, potentially enabling efficient extraction of antioxidant compounds from plants.

##### Ferric Reducing Antioxidant Power (FRAP) Activity

**Figure 6:**
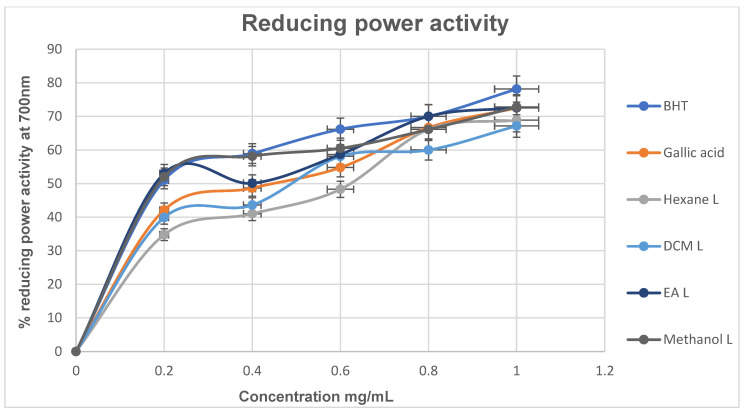
Percentage (%) reducing power ability of *M. balsamina* extracts at different concentrations.

The principle of the ferric reducing antioxidant power (FRAP) assay is based on the ability of the potential antioxidant to reduce the Fe^3+^–2,4,6-tripyridyl-S-triazine (TPTZ) complex to a more stable Fe^2+^ ion under acidic conditions [46]. The reduction in ferricyanide leads to an increase in absorbance, measured spectrophotometrically at 593 nm, which is directly proportional to the reducing power and thus reflects antioxidant potential [47]. Compounds with reducing power act as electron donors, reducing the oxidized intermediates of lipid peroxidation to more stable products and thereby terminating radical chain reactions, which function as both primary and secondary antioxidants [48]. As shown in Figure 6, the standard BHT exhibited a higher reducing power activity than all extracts, with a percentage reducing power of 78.15% at the highest concentration tested (1 mg/mL). This was followed by the polar extracts, ethyl acetate (72.59%) and methanol (72.80%). Notably, at the lowest concentration tested, ethyl acetate (53.05%) and methanol (52.01%) demonstrated superior reducing power compared to the gallic acid (42.12%) and BHT (51.03%) standards, as well as the less polar extracts (hexane 34.80% and DCM 39.91%). These findings highlight the strong reducing potential of polar extracts and reinforce the role of solvent polarity in enhancing the extraction of bioactive compounds with significant antioxidant activity.

The IC_50_ values obtained (Table 5) in this study provide important insight into the antioxidant potential of the extracts in relation to diabetes management, as oxidative stress is a key contributor to the onset and progression of diabetes and its complications. Lower IC_50_ values indicated stronger antioxidant activity, suggesting a greater ability to neutralize reactive oxygen species implicated in pancreatic β-cell dysfunction and insulin resistance. In the DPPH assay, the ethyl acetate (IC_50_ = 0.232 mg/mL) and methanol (0.237 mg/mL) extracts exhibited stronger radical scavenging activity than the standard gallic acid (0.441 mg/mL) and were comparable to BHT (0.304 mg/mL), indicating potent free radical neutralizing capacity. Similarly, in the hydrogen peroxide scavenging assay, the ethyl acetate extract (0.244 mg/mL) demonstrated the strongest activity among all samples, even outperforming both standards, which is particularly relevant since hydrogen peroxide can penetrate biological membranes and contribute to cellular oxidative damage in diabetic conditions.

For reducing power, although BHT (0.439 mg/mL) remained the most potent, the methanol (0.475 mg/mL) and ethyl acetate (0.518 mg/mL) extracts showed comparable activity, suggesting their ability to act as electron donors and terminate oxidative chain reactions. In contrast, the hexane and dichloromethane extracts generally exhibited weaker activity across the assays, reflecting the lower extraction efficiency of antioxidant compounds in less polar solvents. Overall, the strong antioxidant activity observed, particularly in the semi-polar and polar extracts, supports their potential role in solubilizing antioxidant phytochemicals and mitigating oxidative stress associated with diabetes. This further suggests that these extracts may contribute to the protection of pancreatic cells and improvement of glucose metabolism, thereby reinforcing the therapeutic relevance of the plant in diabetes management.

**Table 5:** IC_50_ of various *M. balsamina* leaf and fruit extracts.

Extracts and Standards		IC_50_ (mg/mL)		
		DPPH Scavenging	H_2_O_2_ Scavenging	Reducing Power
Gallic Acid		0.441	0.447	0.540
BHT		0.304	0.401	0.439
Hexane	Leaf	0.565	0.724	0.607
DCM	Leaf	0.259	0.818	0.586
EA	Leaf	0.232	0.244	0.518
Methanol	Leaf	0.237	0.721	0.475

### 3.5. Antidiabetic Activity

The potential of *M. balsamina* extracts to regulate blood glucose levels was assessed through α-amylase, α-glucosidase, and β-glucosidase inhibitory assays. The inhibitory activities of *M. balsamina* extracts were compared to that of acarbose, a standard antidiabetic drug used in the management of type 2 diabetes. Acarbose was selected as a reference inhibitor due to its well-established role in targeting key carbohydrate-metabolizing enzymes [49].

#### 3.5.1. Alpha-Amylase Inhibition Activity

The enzyme α-amylase facilitates the breakdown of polysaccharides into simple sugars, enabling their absorption in the intestine. The inhibition of α-amylase reduces carbohydrate digestion and absorption, thereby helping to regulate blood glucose levels [50]. As a result, α-amylase inhibition is considered a promising approach for managing postprandial hyperglycemia.

**Figure 7:**
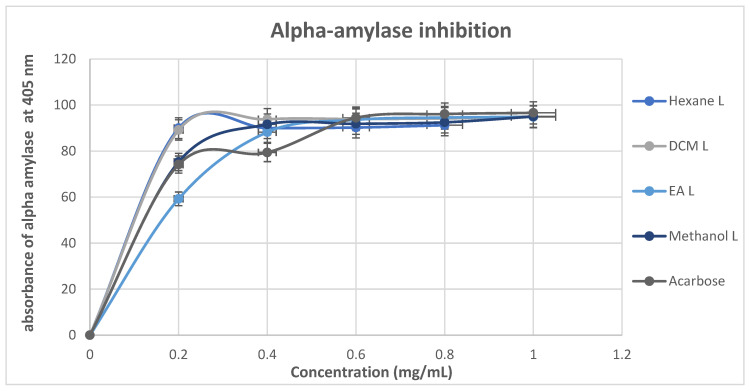
The α-amylase inhibitory activity of *M. balsamina* leaf extracts at different concentrations.

As illustrated in Figure 7, all the *M. balsamina* extracts exhibited an inhibitory pattern resembling that of the standard antidiabetic drug acarbose. The inhibition of α-amylase by *M. balsamina* extracts and acarbose showed an increase in a concentration-dependent manner, from the lowest concentration (0.2 mg/mL) up to a concentration of 0.6 mg/mL where they all reached a state of plateau. The plateau state appeared between the concentrations of 0.6 and 1.0 mg/mL, and all the extracts and standards demonstrated a near-maximum inhibition percentage above 90%. The results imply that *M. balsamina* extracts may control blood glucose levels and efficiently suppress α-amylase activity. The plateau state suggest that maximum inhibition has been reached and that any further increase in the concentration of test will make no difference. At the lowest concentration of 0.2 mg/mL, hexane, DCM, and methanol (89.98%, 89.19%, 75.3) surpassed the inhibition by acarbose, which exhibited a percentage inhibition of 78.14%. Similarly, at the concentration of 0.4 mg/mL, all the extracts (hexane: 90.0%, EA: 88.25%, DCM: 93.83, methanol: 91.59%) exhibited stronger α-amylase inhibition than acarbose (79.41%). These results suggest that *M. balsamina* extracts are potential natural alternatives for controlling postprandial hyperglycemia, and it is a good indication that the compounds exhibit stronger antidiabetic potential at lower concentrations, because excessive α-amylase inhibition has been associated with side effects such as gastrointestinal complications.

#### 3.5.2. Alpha-Glucosidase Inhibitory Activity

α-glucosidase is an enzyme responsible for catalyzing the breakdown of disaccharides into monosaccharides, facilitating their absorption in the small intestine. Inhibitors of α-glucosidase slow the final stages of carbohydrate digestion, thereby preventing the rapid entry of glucose into the bloodstream [51]. Inhibition of this enzyme delays glucose absorption, which helps to stabilize blood glucose levels and thus contribute to the management of diabetes progression.

**Figure 8:**
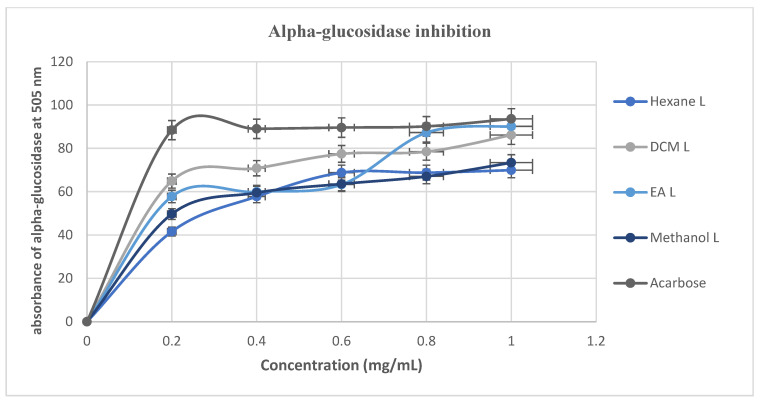
The α-glucosidase inhibitory activity of *M. balsamina* leaf and fruit extracts at different concentrations.

The results for α-glucosidase inhibitory activity in this study are presented in Figure 8, showing that all the extracts and standard followed a concentration-dependent response; that is, the inhibition of the enzyme increases as the concentration of test samples increase. The standard drug acarbose exhibited stronger α-glucosidase inhibition activity than all the *M. balsamina* extracts throughout all the tested concentrations (0.2 to 1.0 mg/mL), with percentages of inhibition ranging from 88.44 to 93.63%. At the lowest concentration of 0.2 mg/mL, DCM (64.91%) exhibited the highest activity, followed by EA (57.8%), methanol (49.71%), and then hexane (41.62%).

#### 3.5.3. Beta-Glucosidase Inhibitory Activity

The inhibitory potential of β-glucosidase was evaluated due to its critical role in carbohydrate digestion and metabolic regulation. The inhibition of this enzyme delays the breakdown of complex carbohydrates into absorbable glucose, thereby reducing postprandial hyperglycemia, a key factor in the management of type 2 diabetes. Additionally, modulation of β-glucosidase activity can influence various metabolic processes, further highlighting its importance as a therapeutic target.

**Figure 9:**
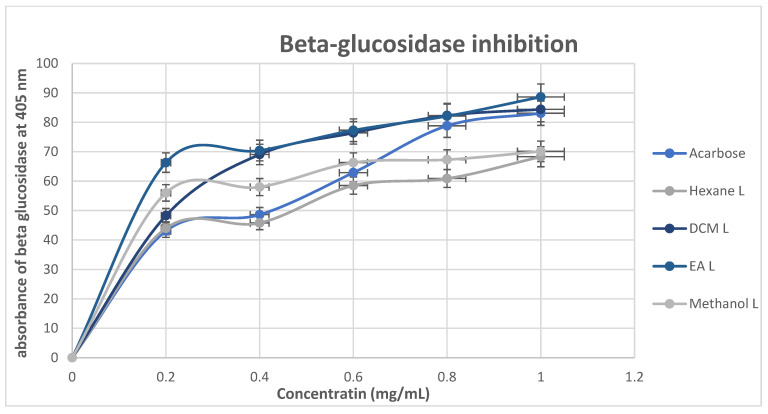
The β-glucosidase inhibitory activity of *M. balsamina* leaf and fruit extracts at different concentrations.

The β-glucosidase inhibitory activity of the *M. balsamina* extracts and the standard drug acarbose, as shown in Figure 9, demonstrated a concentration-dependent increase where the concentration increased from 0.2 to 1.0 mg/mL; all extracts exhibited enhanced inhibitory activity, indicating effective enzyme interaction. At all the concentrations tested, the EA extract showed the highest inhibitory activity of 66.3 to 88.61%. At the lowest tested concentration (0.2 mg/mL), all extracts—hexane (44.0%), DCM (48.29%), ethyl acetate (66.30%), and methanol (56.01%)—exhibited stronger β-glucosidase inhibitory activity than the standard drug acarbose (43.06%). This suggests the presence of potent bioactive compounds with significant enzyme inhibitory potential. Overall, the superior performance of the semi-polar EA extracts suggested that the compounds responsible for β-glucosidase inhibition are likely enriched in this fraction, supporting their potential use in the management of postprandial hyperglycemia.

#### 3.5.4. IC_50_ Values of Enzymes and Standard Against *Momordica balsamina* Linn. Extracts

The half maximal inhibitory concentration (IC_50_) values of all the *M. balsamina* extracts and acarbose for α-amylase inhibition were determined (Table 6). All extracts displayed lower IC_50_ values (0.106–0.204 mg/mL) compared to acarbose (IC_50_ = 0.209 mg/mL), with the DCM extract exhibiting the lowest IC_50_ (0.106 mg/mL). The lower IC_50_ values indicated that *M. balsamina* extracts exhibited considerable α-amylase inhibitory activity, suggesting their potential as effective antidiabetic agents that may perform comparably or even better than existing pharmaceutical treatments like acarbose. The IC_50_ values for α-glucosidase, calculated and presented in Table 6, revealed that acarbose exhibited the lowest IC_50_ value of 0.129 mg/mL as compared to *M. balsamina* extracts, which ranged from 0.31 to 0.533. Among the extracts, the DCM extract showed the lowest IC_50_ value of 0.31, followed by the EA extract (0.374). While the differences in IC_50_ values between acarbose and the extracts may not be significant, the results suggested promising inhibitory activity of the extract. Furthermore, the isolation of active compounds is warranted, as the antidiabetic effects of *M. balsamina* may be attributable to a specific compound or a synergistic combination of bioactive constituents. The IC_50_ of acarbose, a well-known pharmacological inhibitor of β-glucosidase enzymes, was 0.464 mg/mL. Both the DCM (IC_50_ of 0.367 mg/mL) and EA leaf (IC_50_, 0.301 mg/mL) extracts exhibited lower IC_50_ values than acarbose, suggesting they may have similar or even better inhibitory effects. These results suggest that the plant extracts may have therapeutic value, especially if they also have a good safety record.

Overall, the results demonstrated that *M. balsamina* possesses multi-target enzyme inhibitory activity, with particularly strong effects against α-amylase and β-glucosidase. The consistent performance of the DCM and EA extracts across assays suggested that solvent polarity plays a key role in extracting potent bioactive compounds. Although acarbose remains more effective against α-glucosidase, the comparable or superior activity of the plant extracts in other assays highlighted their therapeutic potentials. 

**Table 6:** The IC_50_ values of the standard and various *M. balsamina* extracts.

Standard and Extract Samples	IC_50_ (mg/mL)		
	α-Amylase	α-Glucosidase	β-Glucosidase
Acarbose	0.209	0.129	0.404
Hexane	0.206	0.475	0.565
DCM	0.149	0.310	0.367
EA	0.146	0.374	0.301
Methanol	0.176	0.463	0.447

IC_50_, half maximal inhibitory concentration.

### 3.6. Cytotoxicity

**Figure 10:**
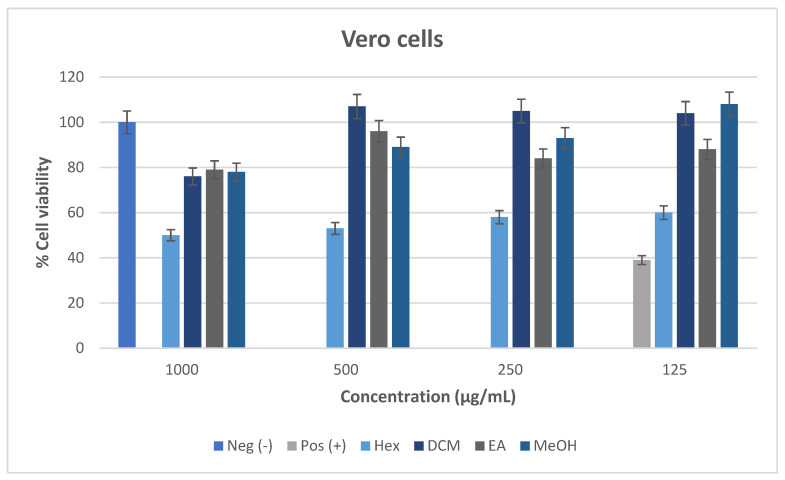
Cell survival following treatment of Vero cells for 48 h with *M. balsamina* leaf extracts. Data represent the mean ± standard deviation of two independent experiments.

**Figure 11:**
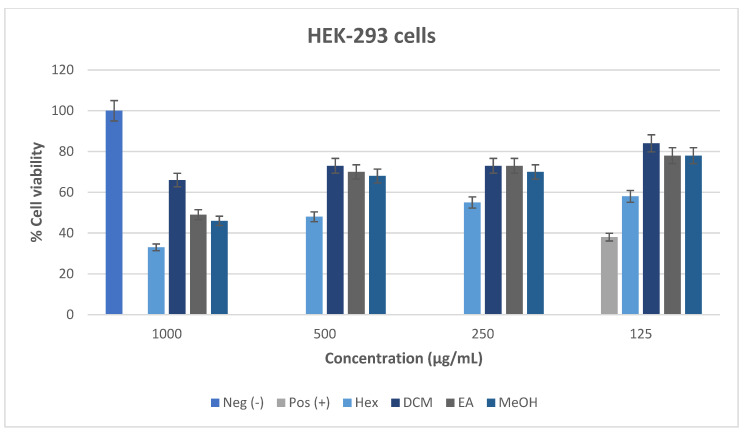
Cell survival following treatment of HEK-293 for 48 h with *M. balsamina* leaf extracts.

The cytotoxic effect of *M. balsamina* leaf extracts was assessed using the MTT assay against the Vero and HEK-293 cell lines, as illustrated in Figure 10 and Figure 11. The MTT assay was based on the ability of mitochondrial dehydrogenases in viable cells to reduce MTT, converting it from a yellow tetrazolium salt into insoluble purple formazan. The cytotoxic effect of the test samples can be classified based on the proportion of viable cell as follows: >80% = non-cytotoxic; 60–80% = weakly cytotoxic; 40–60% = moderately cytotoxic; and <40% = highly cytotoxic [52].

#### 3.6.1. Cytotoxicity of *M. balsamina* Extracts Against Vero Cell Lines

The cytotoxic effect of *M. balsamina* extracts against the Vero cells in Figure 10 demonstrated a dose-dependent response, whereby, percentage cell viability increases as the test concentration decreases. The negative control, consisting of untreated cells, maintained 100% viability, confirming normal cell growth and validating the assay. In contrast, the positive control, hydrogen peroxide, exhibited the lowest cell viability of below 40% at the lowest concentration tested (125 µg/mL), indicated strong cytotoxicity and confirming the sensitivity of the assay system. The DCM, EA, and MeOH extracts exhibited percentage cell viability above 60% in all the concentrations tested (125 to 1000 µg/mL), and underscored that they were not toxic to the cells. Hexane extract, on the other hand, demonstrated the lowest cell viability in all the concentrations tested with a percentage viability below 40%, indicating pronounced cytotoxicity. The findings suggested that DCM, EA, and MeOH extracts, as well as the semi-polar and polar extracts, were generally less toxic than the non-polar hexane extract, demonstrating that cytotoxic constituents were likely concentrated in the non-polar fractions than the polar ones. Overall, the results suggest that the semi-polar and polar extracts are non-toxic and may be considered safe for use under the tested conditions. 

Based on the observed viability values, the IC_50_ (the concentration required to reduce cell viability by 50%) could not be reached within the tested concentration range for all the extracts, as cell viability remained well above 50%. Therefore, the IC_50_ values of the extracts were estimated to be greater than the highest concentration tested, further supporting their low cytotoxic potential.

#### 3.6.2. Cytotoxicity of *M. balsamina* Extracts Against HEK-293 Cell Lines

The cytotoxic effect of *M. balsamina* extracts, as illustrated in Figure 11, demonstrated a clear dose-dependent reduction in cell viability of the HEK-293 cell lines. At the highest concentration (1000 µg/mL), the hexane extract exhibited the lowest cell viability, indicating pronounced cytotoxicity, while the dichloromethane (DCM), ethyl acetate (EA), and methanol (MeOH) extracts showed moderate toxicity. As the concentration decreased, all extracts demonstrated improved cell viability, with DCM consistently showing the highest viability across all concentrations, suggesting lower toxicity and better biocompatibility. The EA and MeOH extracts also maintained relatively stable and moderate to high viability, particularly at 250 and 125 µg/mL. In contrast, the hexane extract remained the most cytotoxic across all tested concentrations, although a gradual improvement in viability was observed at lower doses. Notably, none of the extracts exceeded 100% cell viability, indicating an absence of proliferative effects in HEK-293 cells compared to Vero cells. Overall, these findings suggest that semi-polar and polar extracts (DCM, EA, and MeOH) exhibit lower cytotoxicity, while the non-polar hexane extract contains compounds that may contribute to increased toxicity, highlighting the relative safety of the former for further biological applications.

## 4. Conclusions

The current study demonstrated the noteworthy in vitro antidiabetic activity of *Momordica balsamina* L. leaf extracts, as evidenced by their ability to inhibit α-amylase, α-glucosidase, and β-glucosidase. The observed effects may be attributed to the presence of bioactive compounds identified through GC–MS analysis, many of which have been previously reported to possess antidiabetic properties. Among the extracts, ethyl acetate (EA) and dichloromethane (DCM) fractions exhibited the strongest enzyme inhibitory activities, coupled with non-cytotoxic responses in the MTT assay against Vero cell lines, indicating favorable safety profiles and potential for therapeutic applications.

In South Africa, *M. balsamina* is widely used in traditional medicine for the management of diabetes and is, in some cases, recommended by healthcare practitioners. The findings of this study therefore provide scientific validation for its ethnomedicinal use and highlights its promise as a source of plant-based antidiabetic agents. However, further studies are required to isolate and characterize the active compounds and elucidate their mechanisms of action, and confirm their efficacy and safety through in vivo and pharmacokinetic investigations to support future drug development using non-carcinogenic solvent, like ethyl acetate or ethanol as recommended extractants.

## Figures and Tables

**Table 2:** Total phenolic and flavonoid contents in *Momordica balsamina* Linn leaf extracts.

Phytochemicals Evaluated	Mean of Concentrations in μg/mL of Extracts of Different Polarities ± Standard Deviation			
	Hexane	Dichloromethane	Ethyl Acetate	Methanol
TPC (µg GAE/g extract)	10.532 ± 1.663	16.553 ± 1.221	9.812 ± 1.003	10.99 ± 3.442
TFC (µg QE/g extract)	2.338 ± 0.282	2.111 ± 0.343	3.661 ± 0.008	4.889 ± 0.060

## Data Availability

The original contributions presented in this study are included in the article. Further inquiries can be directed to the corresponding authors.

## Abbreviations

The following abbreviations are used in this manuscript:

AMPK	Adenosine Monophosphate-Activated Protein Kinase
BHT	Butylated Hydroxytoluene
DCM	Dichloromethane
DMEM	Dulbecco’s Modified Eagle Medium
DPPH	2,2-Diphenyl-1-Picrylhydrazyl
EA	Ethyl Acetate
FRAP	Ferric Reducing Antioxidant Power
GC-MS	Gas Chromatography–Mass Spectrometry
H_2_O_2_	Hydrogen Peroxide
HEK-293	Human Embryonic Kidney 293 Cells
IC_50_	Half Maximal Inhibitory Concentration
IRS1	Insulin Receptor Signaling 1
IRS	Insulin Receptor Substrate
LC-MS	Liquid Chromatography–Mass Spectrometry
*M. balsamina*	*Momordica balsamina*
MeOH	Methanol
Min	Minutes
MTT	3-(4,5-Dimethylthiazol-2-yl)-2,5-Diphenyltetrazolium Bromide
PI3K	Phosphatidylinositol 3-Kinase
PTP1B	Protein Tyrosine Phosphatase 1B
ROS	Reactive Oxygen Species
RT	Retention Time
SANBI	South African National Biodiversity Institute
